# Contrasting effects of linezolid on healthy and dysfunctional human neutrophils: reducing C5a-induced injury

**DOI:** 10.1038/s41598-020-72454-0

**Published:** 2020-10-02

**Authors:** Stephen J. Evans, Aled E. L. Roberts, Andrew Conway Morris, A. John Simpson, Llinos G. Harris, Dietrich Mack, Rowena E. Jenkins, Thomas S. Wilkinson

**Affiliations:** 1 Microbiology and Infectious Disease, Institute of Life Science, Swansea University Medical School, Floor 1, Room 137, Singleton Park, Swansea, SA2 8PP UK; 2 Division of Anaesthesia, Department of Medicine, School of Clinical Medicine, University of Cambridge, Level 4, Addenbrooke’s Hospital, Cambridge Biomedical Campus, Hills Road, Box 93, Cambridge, CB2, 0QQ UK; 3 Institute of Cellular Medicine, Medical School, Newcastle University, Newcastle upon Tyne, NE2 4HH UK; 4 Bioscientia Labor Ingelheim, Institut für Medizinische Diagnostik GmbH, Konrad-Adenauer-Str. 17, 55218 Ingelheim, Germany

**Keywords:** Infectious diseases, Respiratory tract diseases, Cell biology, Microbiology, Infectious diseases, Innate immune cells, Inflammation, Acute inflammation

## Abstract

Methicillin-resistant *Staphylococcus aureus* (MRSA) is an important cause of ventilator-associated pneumonia (VAP). Patients with VAP have poorly functioning neutrophils, related to increased levels of the complement fragment C5a. The antibiotic linezolid has been useful in controlling MRSA-related VAP infections; however clinical benefit does not always correlate with antimicrobial effect, suggesting the possibility of immunomodulatory properties. Here the effects of linezolid on healthy and dysfunctional neutrophils (modelled by C5a-induced injury) was investigated. Functional assays (killing, phagocytosis, transmigration, and respiratory burst) were used to assess the effects of pre-, co- and post-incubating linezolid (0.4–40 mg/L) with healthy neutrophils relative to those with C5a-induced injury. C5a decreased neutrophil killing, and phagocytosis of MRSA. Furthermore, C5a significantly decreased neutrophil transmigration to IL-8, but did not affect respiratory burst. Co-incubation of linezolid significantly improved killing of MRSA by dysfunctional neutrophils, which was supported by concomitant increases in phagocytosis. Conversely linezolid impaired killing responses in healthy neutrophils. Pre- or post-incubation of linezolid prior or following C5a induced injury had no effect on neutrophil function. This study suggests that linezolid has immunomodulatory properties that protect human neutrophils from injury and provides insight into its mode of action beyond a basic antibiotic.

## Introduction

Ventilator-associated pneumonia (VAP) is an important infection acquired in the intensive care unit (ICU) and can occur in up to 20% of patients mechanically ventilated for periods greater than 48 h^1^. The 2016 annual European report on healthcare-associated infections showed that of 12,735 patients staying more than 2 days in ICU, 6% developed pneumonia (where 97% of these were intubated), with *Staphylococcus aureus* the causative organism in 17.8% of cases (30% being methicillin-resistant *S. aureus* (MRSA))^2^. Treatments for MRSA pneumonia have relied on vancomycin and teicoplanin until the introduction of linezolid in 2000, which has been particularly successful at treating MRSA pneumonia^3–5^. While it is clear that linezolid has a clinical^3–5^ and economic benefit^6,7^ in treating MRSA pneumonia, the apparent treatment effect is not due to its antimicrobial activity alone^3,8^, suggesting potential host-specific effects, such as immunomodulation.

The underlying illness in critically ill patients, including those with VAP, is often associated with major deficiencies in the innate immune system^9^ specifically, neutrophil dysfunction, an inability to phagocytose or produce a respiratory burst^10,11^. Our previous studies found that VAP patients’ neutrophils had 36% lower phagocytic capacity than healthy volunteers, and was significantly and negatively correlated with serum C3a des-Arg (C3a breakdown product) and positively correlated with neutrophil cell surface expression of the C5a receptor (CD88). In vitro modelling in healthy volunteer neutrophils found that C5a treatment could mimic impaired phagocytosis and down-regulate CD88^12–14^.

The effect of linezolid on dysfunctional human neutrophils has not been studied to date. However, numerous studies have addressed the effects of linezolid on neutrophils from healthy volunteers. For instance, linezolid at concentrations of 10–160 mg/L, had no negative effects on chemotaxis or respiratory burst in either a pure drug or intravenous injection formulation^15^. Similarly, pre-incubation of healthy neutrophils with linezolid (2–20 mg/L) had no effect on phagocytosis of methicillin-resistant or -susceptible *S. aureus*, or *Enterococcus faecalis* (vancomycin-resistant or -susceptible)^16^. Other studies have shown small decreases in phagocytosis in response to certain strains of *Escherichia coli*^17^. Interestingly, Pascual and co-workers have shown that linezolid penetrates the neutrophil rapidly as intracellular concentrations greater than those of the external environment are reached within 20 min^18^. This ability to cross biological membranes is reflected in its high concentrations in lung epithelial lining fluid (ELF). These studies suggest that linezolid in concentrations far in excess of its MIC (4 mg/L)^19^ are not cytotoxic to healthy neutrophils using a variety of functional assays.

Using our established model of C5a-induced neutrophil dysfunction^12,13^, we investigated the effect of linezolid on both healthy and clinically relevant C5a-impaired neutrophils in a variety of relevant functional assays.

## Results

**Figure 1:**
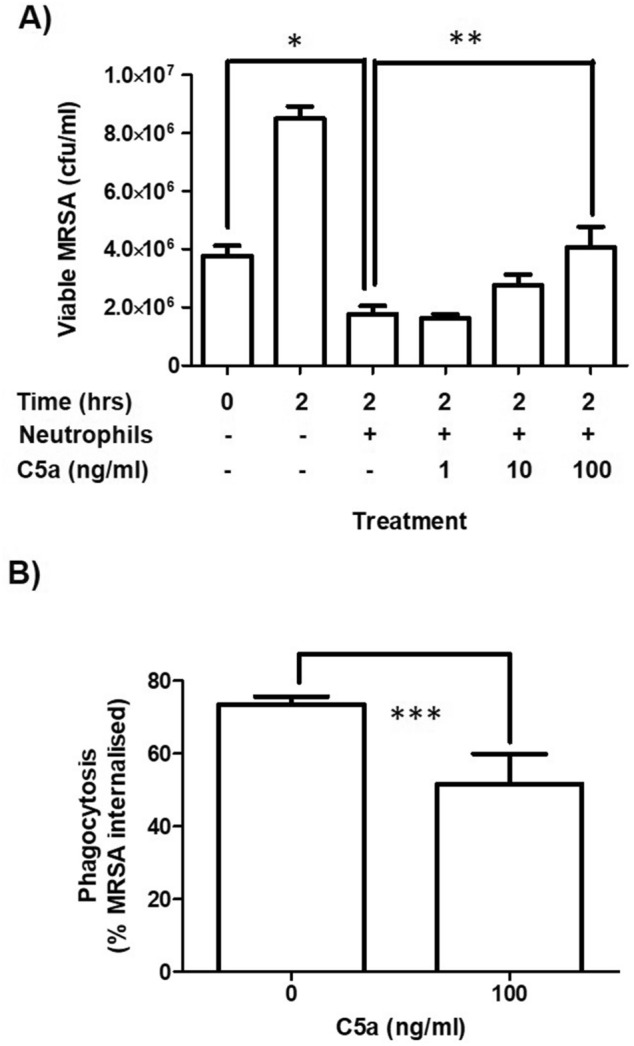
Effects of C5a on neutrophil function: killing and phagocytosis. Purified neutrophils were treated with or without C5a (1–100 ng/ml) for 16 h prior to functional assay. (**A**) Killing expressed as viable counts of MRSA (cfu/ml). (**B**) Phagocytosis expressed as the percentage of neutrophils containing MRSA. Data are expressed as the mean ± SEM of 4 separate donors. *Represents a significant difference between viable counts of MRSA at t = 0 versus t = 2 with neutrophils present. **Represents a significant difference between viable counts of MRSA at t = 2 with neutrophils present versus t = 2 with neutrophils present treated with C5a at 100 ng/ml. ***Represents a significant difference in phagocytosis between neutrophils treated and not treated with C5a.

Killing response of neutrophils to VAP39 resulted in ~ 50% decrease in colony counts over the first 2 h of infection (Fig. 1A). MRSA incubated without neutrophils doubled in number over the same period showing strain viability in our assay media. Incubation of neutrophils with increasing doses of C5a (1–100 ng/ml) resulted in a significant increase in the survival of MRSA at the highest dose of C5a (100 ng/ml) demonstrating dysfunctional killing responses. Consistent with this result a significant decrease (*p* < 0.05) was detected in neutrophil phagocytosis of VAP39 (Fig. 1B and Supplementary Image 2) over 2 h. Thus C5a (100 ng/ml) decreased neutrophil killing which is associated with attenuated phagocytosis (Fig. 1A,B).

**Figure 2:**
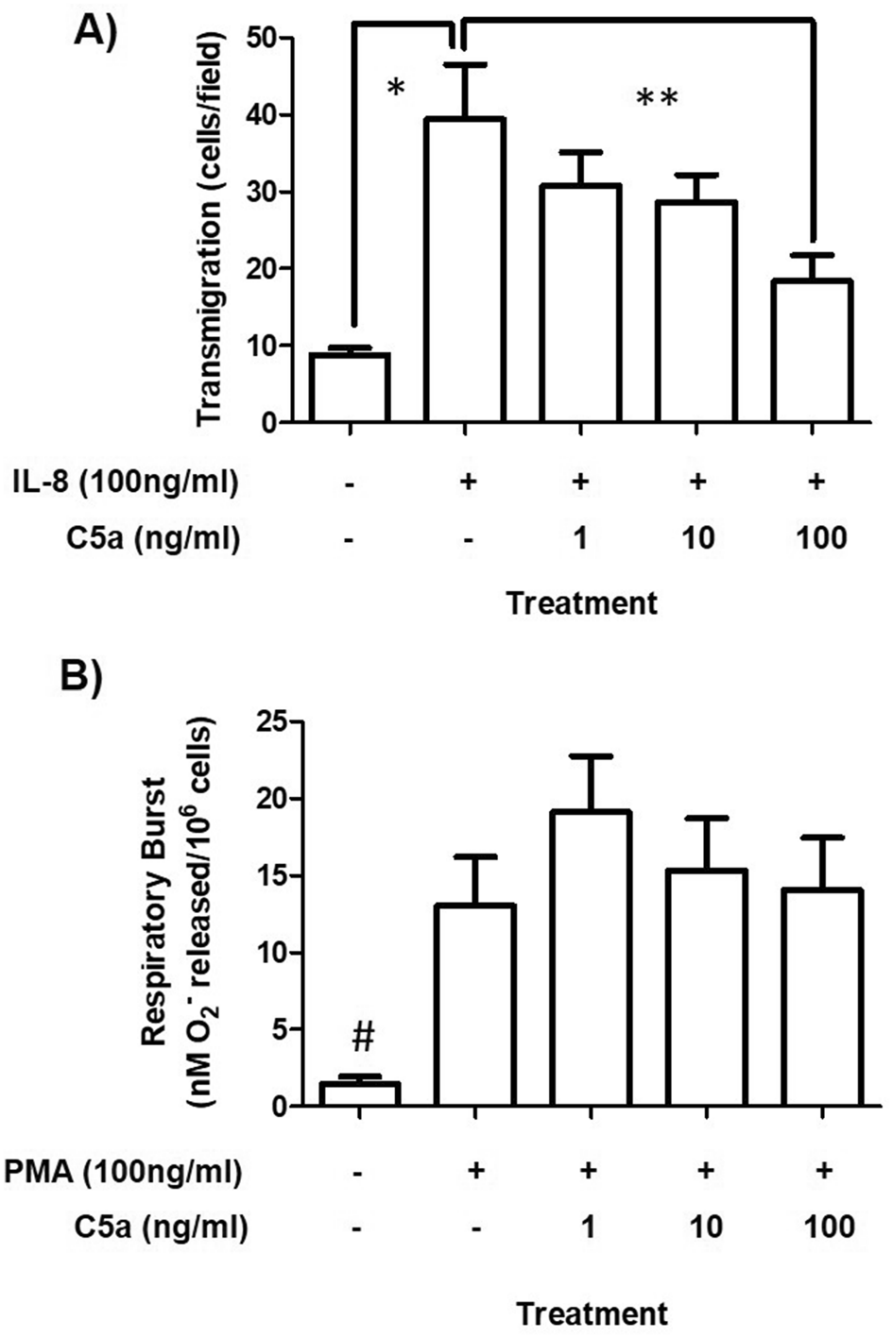
Effects of C5a on neutrophil function: transmigration and respiratory burst. (**A**) Transmigration is expressed as the number of cells counted per high power field. (**B**) Respiratory burst is expressed as the amount of superoxide release per million cells. Data are expressed as the mean ± SEM of 4 separate donors. Differences between groups were calculated using ANOVA with a Tukey’s post-hoc test where *p* < 0.05 considered significant. *Represents a significant difference between neutrophil transmigration with and without IL-8. **Represents a significant difference between IL-8 stimulated neutrophils treated with and without C5a. #Represents a significant difference in respiratory burst between neutrophils stimulated with PMA and those without PMA.

The effect of C5a on the ability of neutrophils to transmigrate across membrane filters in response to IL-8 was investigated (Fig. 2A). IL-8 induced a significant increase (*p* < 0.05) in neutrophil transmigration (Fig. 2 and Supplementary image 3). Addition of C5a caused a dose-dependent decrease in IL-8-induced transmigration which reached significance (*P* < 0.05) at the highest dose of C5a (100 ng/ml). In contrast, while neutrophil respiratory burst (Fig. 2B) was significantly induced (*p* < 0.05) using PMA it was independent of C5a over the dose range studied. Collectively these results (Figs. 1 and 2) demonstrate that functional responses (without C5a) and dysfunctional responses (with C5a at 100 ng/ml) can be produced in killing, phagocytosis and transmigration. Neutrophils are viable as confirmed by full respiratory burst in healthy and dysfunctional neutrophils (Fig. 2B). Neutrophil metabolic activity measurements using alamar blue confirmed that cellular reducing power decreased up to 10% and 45% in functional and dysfunctional neutrophils respectively (data not shown) and is consistent with a recent study confirming the effects of C5a on cellular respiration^20^. Using these two conditions (with and without C5a at 100 ng/ml) the effect of linezolid on functional and dysfunctional neutrophils was investigated.

**Figure 3:**
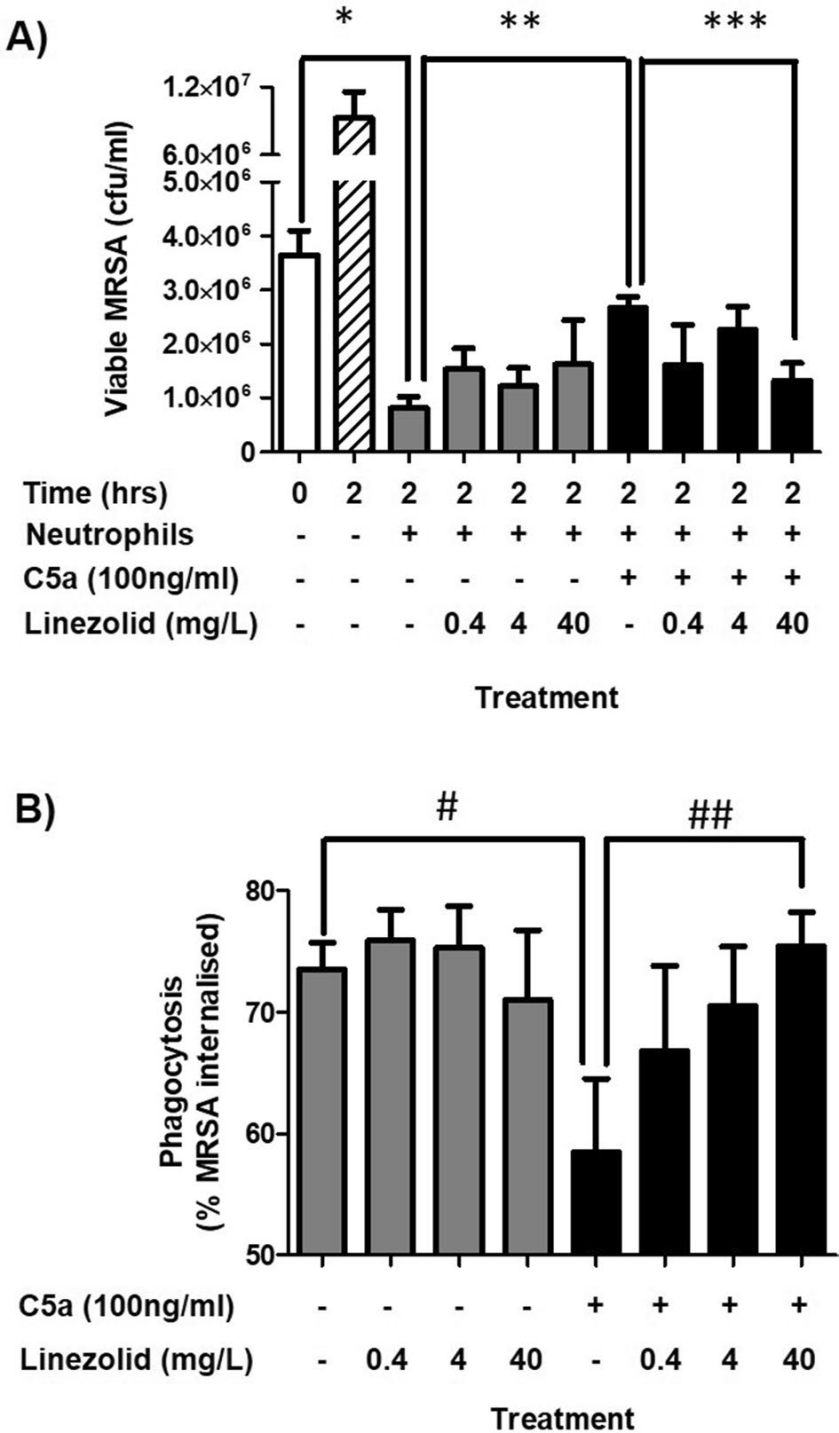
Effects of linezolid on functional and dysfunctional neutrophil killing and phagocytosis. (**A**) Killing expressed as viable counts of MRSA (cfu/ml). (**B**) Phagocytosis expressed as the percentage of neutrophils containing MRSA. Data are expressed as the mean ± SEM of 4 separate experiments. White bar represents viable MRSA at t = 0. Hatched bar represents viable MRSA at t = 2 without neutrophils. Grey and black bars represent neutrophils at t = 2 without and with C5a respectively. *Represents a significant difference between viable counts of MRSA at t = 0 versus t = 2 with neutrophils present. **Represents a significant difference between viable counts of MRSA treated with functional and dysfunctional neutrophils at t = 2. ***Represents a significant difference between viable counts of MRSA exposed to dysfunctional neutrophils treated with or without linezolid. # Represents a significant difference in phagocytosis of MRSA by functional and dysfunctional neutrophils. ## Represents a significant difference in the phagocytosis of MRSA by dysfunctional neutrophils with or without linezolid.

Functional (grey bars) and dysfunctional neutrophils (black bars) were generated and co-incubated with linezolid during the C5a incubation period (Fig. 3). Functional neutrophils killed MRSA over 2 h (white bar vs. first grey bar in Fig. 3A), whereas linezolid had no effect on killing by functional neutrophils (Fig. 3A, grey bars). This result also confirms there was no anti-bacterial effect from linezolid carried over inside neutrophils. In contrast, linezolid significantly improved (at 40 mg/L) killing of VAP39 in dysfunctional neutrophils (Fig. 3A, black bars). Indeed, the killing response was no different to that produced initially in functional neutrophils (first grey bar vs. final black bar) showing that linezolid could prevent C5a-induced injury in neutrophils when co-incubated with C5a. Further investigation confirmed that linezolid, co-incubated with C5a could also attenuate the defect induced in phagocytosis of MRSA (Fig. 3B). Thus, linezolid appears to improve killing by dysfunctional neutrophils by improving phagocytosis. This mechanism was only observed during co-incubation and not when linezolid was incubated prior to or after the C5a-injury period (Supplementary Figs. 2 and 3). Further assessment of neutrophil metabolic activity by alamar blue confirmed that linezolid decreased (10–20%) the reducing power of neutrophils after 1 h (pre and post-incubation protocols) and by 10–30% over 16 h (co-incubation) (Supplementary Fig. 4) when given alone. In the absence and presence of C5a, the reducing power of the cell decreased 10 and 50% respectively and is consistent with a recent study confirming the effects of C5a on cellular respiration^20^.

**Figure 4:**
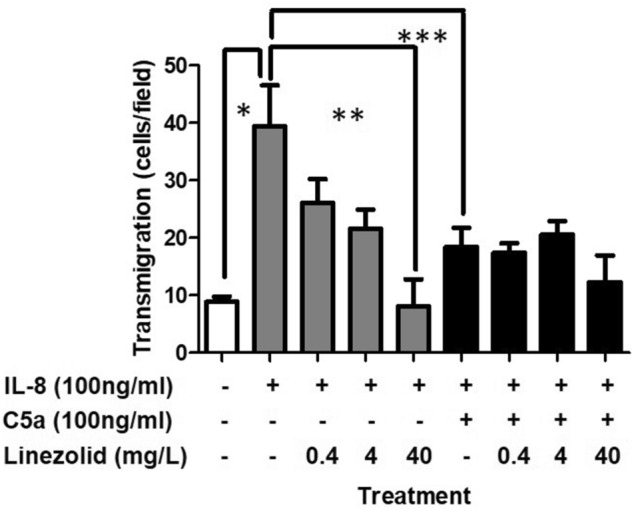
Effects of linezolid on functional and dysfunctional neutrophil IL-8 induced transmigration. Grey and black bars represent functional and dysfunctional neutrophils, respectively. Data expressed as the mean ± SEM of 4 separate experiments. *Represents a significant difference between the transmigration of functional and dysfunctional neutrophils. **Represents a significant difference between the transmigration of neutrophils treated with linezolid (40 mg/L) and those without.

The effect of linezolid on IL-8-induced transmigration was then assessed by co-incubating functional and dysfunctional neutrophils with linezolid during C5a incubation period (Fig. 4). In functional neutrophils, linezolid caused a significant dose dependent decrease in transmigration (Fig. 4, grey bars), while having no effect on the transmigration of dysfunctional neutrophils (Fig. 4, black bars). Furthermore, pre-incubation or post-incubation with linezolid prior or after the C5a injury period had no significant effects on transmigration, (Supplementary Fig. 5A and B).

**Figure 5:**
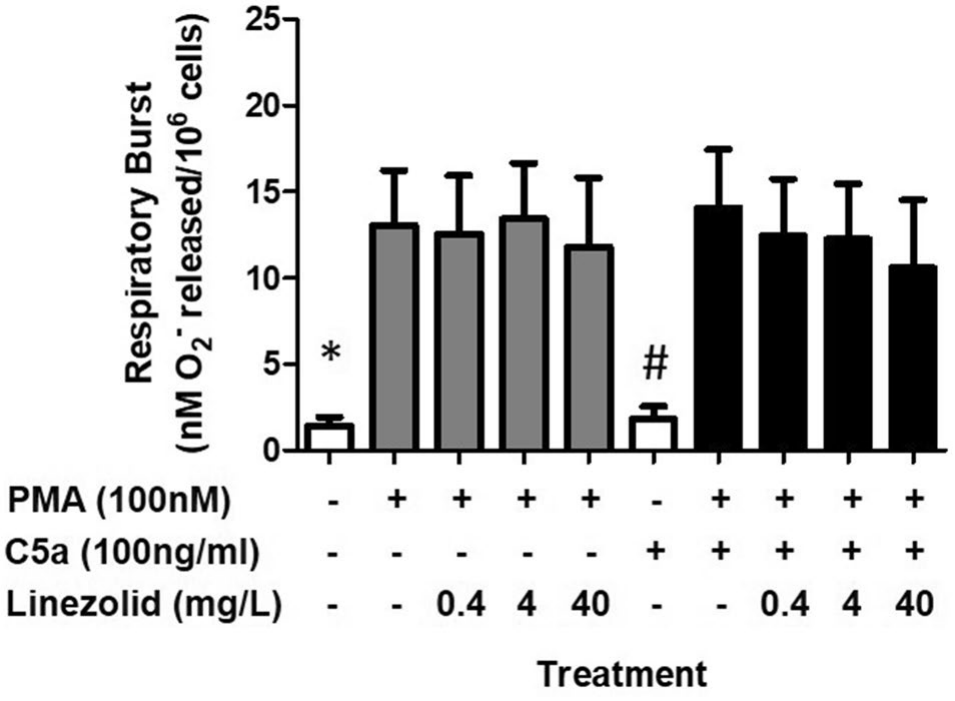
Effects of linezolid on functional and dysfunctional neutrophil respiratory burst. Grey and black bars represent functional and dysfunctional neutrophils, respectively following treatment with PMA and linezolid. Data expressed as the mean ± SEM of 4 separate experiments. *Represents a significant difference between PMA treated and untreated functional neutrophils (grey bars). # Represents a significant difference between PMA treated and untreated dysfunctional neutrophils (black bars).

Finally, the effects of linezolid on respiratory burst (Fig. 5) were assessed by adding PMA to functional and dysfunctional neutrophils respectively. A significant respiratory burst response to PMA could be generated in functional (Fig. 5, grey bars) and in dysfunctional neutrophils (Fig. 5, black bars) but linezolid had no effect on this PMA induced response (Fig. 5). Samples that were not stimulated by PMA but included combinations with and without C5a or linezolid were not significantly increased compared to negative control in Fig. 5. The fact that a full respiratory burst could be induced in dysfunctional neutrophils, albeit independent of linezolid, encouraged us to investigate whether pre-incubation and post-incubation of linezolid prior to and after C5a induced injury may be affected (Supplementary Fig. 6). The results showed increased trends in respiratory burst at lower doses of linezolid (0.4 and 4 μg/ml) but not at the highest dose (40 mg/L) for both functional and dysfunctional neutrophils.

## Discussion

The current study extends our previous model of C5a-induced dysfunction in response to *P. aeruginosa*^12,13^ and *S. epidermidis* biofilm accumulation in whole blood^21^ to investigate the potential of the oxazolidinone, linezolid, to modulate important innate immune responses in healthy and dysfunctional neutrophils. Concentrations of C5a measured in BAL during lung infection range from 20 to 180 ng/ml^22,23^. The current study used 1–100 ng/ml C5a to induce dysfunction in MRSA killing, phagocytosis and transmigration, but retain a functional respiratory burst. This is in keeping with our previous work on *S. epidermidis* biofilm where C5a concentrations of up to 80 ng/ml were generated *ex vivo*^21^. Taken together our current and previous neutrophil killing and phagocytosis results using *P. aeruginosa*, *S. aureus* and *S. epidermidis* suggest that neutrophil sensitivities to clinically relevant C5a concentrations may well be dependent on bacterial *species* when investigating killing and phagocytosis responses.

There is now very clear scientific evidence, defining the metabolic and signalling pathways underlying functional neutrophils^24^ and those subjected to C5a-induced dysfunction^12,13,20,25^. Thus, ligation of C5aR1 (CD88) by C5a, activates its inherent G-protein activity resulting in phosphoinositide 3-kinase delta (PI3Kδ) activation inhibiting activation of the small GTPase RhoA, actin polymerisation and phagocytosis^13^. At the time of writing Wood and co-workers confirmed that C5a induced decreases in the phagocytosis of *S. aureus* are associated with impaired phagosomal maturation by phosphoproteomic remodelling through selective impairment of phagosomal protein phosphorylation, including endosomal marker ZFYVE16 and V-ATPase protein channel component ATPV1G1^25^. In addition, Denk and co-workers proposed that C5aR1 ligation also leads to selective activation of the Na^+^/H^+^ exchanger causing increased intracellular alkalinisation (pHi), increased glycolytic flux, glucose uptake and lactate and proton excretion^20^. In the current study, alamar blue assessment of the reducing power of the cells also confirmed 50% decreased in response to C5a. This is consistent with broad metabolic defects observed in immune cells (immunoparalysis) and characterised by a switch from oxidative phosphorylation to aerobic glycolysis^26^. Indeed, a deficit in energy status (nutrition delivered—energy expenditure) has been reported as a risk factor for the development of *S. aureus* VAP^27^. These effects also appear reversible, as GM-CSF can reverse the effects of C5a-induced injury by reactivating RhoA at the cellular level^13^ and can improve neutrophil phagocytosis in critically ill patients^28^. Furthermore, others have shown that IFN-γ can partially restore function in immunoparalysed leukocytes^26^.

The present study showed that C5a has little effect on oxygen radical killing mechanisms, unlike a study by Huber–Lang which demonstrated C5a-induced impairment of ROS production and failure of NADPH oxidase assembly^29^. Indeed our previous work could not demonstrate C5a impairment of ROS either, but did find that it could be negatively correlated with C3a des-Arg (C5a degradation product) levels in the serum of critically ill patients^12,13^. This may be attributed to functional differences in rat and human neutrophils. Clearly, the C5a effect we observe is independent on the method of superoxide radical production. In our previous study, neutrophils were primed with 100 nM platelet-activating factor (PAF) and stimulated with 100 nM formyl methionyl leucyl phenylalanine (fMLP), whilst in the current study a single PMA induction was used. Alternatively, killing mechanisms in the current study may be protease-mediated, as we have shown previously for neutrophil dysfunction in the lung^30^.

Choosing appropriate doses of linezolid was important during experimental design of this study. There were three major considerations behind our rationale; firstly, to approximate cellular exposure such as in the epithelial lining fluid (ELF), intracellularly and serum concentrations in humans; secondly, to take into account antibiotic concentrations in health and disease; and thirdly to consider defined MIC breakpoints for antibiotic and pathogen. Numerous pharmacokinetic studies and reviews have addressed these issues^31–34^. For example pharmacokinetic studies in humans confirmed that 600 mg of oral linezolid resulted in mean concentrations of 7.6, 24.3 and 1.4 mg/L 12 h later in plasma, ELF and alveolar cells respectively^31^. The EUCAST breakpoint for linezolid on MRSA is 4 mg/L^19^. Thus, our decision to use linezolid at 0.4, 4 and 40 mg/L was well justified as these concentrations are very likely to contact neutrophils in both injured and uninjured lung.

Three linezolid incubation conditions were used to investigate effects on functional and dysfunctional neutrophils and included, pre-, co-, and post C5a incubation period strategies. The rationale being that protection, inhibition and restoration of neutrophil function could be measured respectively. The current study showed very clearly that linezolid’s effects were produced solely using the co-infection strategy. Thus, speculation on linezolid’s targets must draw evidence from C5a signalling processes and emphasise the importance of the C5aR1 (CD88) cell surface receptor, the PI3Kδ-RhoA pathway, phagosome maturation and modulators of glycolytic flux such as glucose transporters and pHi^12,13,20,25^. Critically, Wood et al. alluded to the timing of exposure to C5a as important in downstream effects^25^. They find that C5a only impairs phagocytosis if the cells are exposed to it before they encounter *S. aureus*—not at the same time or after initial exposure. This effect is independent of CD88. In the current study it seems reasonable to assume that co-infection with linezolid must influence this critical C5a signalling period and its effect is independent of cell surface CD88, but dependent on effects produced intracellularly on pHi or maturation of the phagosome^20,25^.

Further insight into the cellular effects of linezolid may be gained when viewed anatomically. There is good evidence for linezolid penetration into the intracellular compartment of cells^33^, thus binding and biological activity at the level of organelles is likely. Interestingly, sufficient patient data now exist on the inhibitory effect of linezolid on protein synthesis in the mitochondria leading to anaerobic glycolysis and cellular acidosis^35–38^. Specifically with respect to the neutrophil, the mitochondrion has evolved a specific function in apoptosis (not ATP production) and contains numerous apoptotic proteins^39^. There is an intriguing possibility for linezolid to modify neutrophil function in a state-dependent (function/dysfunction) manner as shown in this work. However a recent study by Akinnusi and co-workers could not show any effect of linezolid on neutrophil apoptosis in a MRSA pneumonia mouse model^40^.

In healthy neutrophils, linezolid produced a significant dose-dependent decrease in transmigration. This is partly supported by other studies which showed either no effect on neutrophil functions^15,16^ or mild toxic responses in phagocytosis^17^ over similar dose ranges (0–160 mg/L) to our study (0.4–40 mg/L). The trends towards enhanced respiratory burst observed in this study may be due to the much shorter incubation times (1 h) which may generate a priming effect as with a PAF/fMLP combination shown previously^12^. One strength of the current study is extension of this work to dysfunctional cells which are more likely to be present in the critically ill patient who receives linezolid. Two major findings are particularly pertinent; (1) linezolid inhibited C5a-induced dysfunction in neutrophil killing; (2) linezolid inhibited C5a induced dysfunction in neutrophil phagocytosis. Firstly, these results suggest that the effects of C5a are reversible. Secondly, they support previous observations in clinical studies, where clinical benefit could not be explained by antimicrobial activity alone^3,8^. We speculate that linezolid’s inhibition of C5a-induced dysfunction (under co-incubation conditions only) could be responsible for a large proportion of the underlying mechanism on clinical benefit. Thus, less neutrophils would become dysfunctional in the presence of C5a. Indeed, if this were the case the inhibitory effect that linezolid has on functional neutrophil transmigration may now come into play and also reduce the excessive inflammation seen in the lung during pneumonia. This is compelling as there are already numerous reports that suggest linezolid modulates cytokine cascades^41–46^. Furthermore, such effects on healthy and dysfunctional neutrophils raise the interesting novel possibility that linezolid’s action could be dependent on the state of the cell. This is analogous to antibiotics having activity on dividing cells but not on dormant bacteria^47^.

There is a growing literature that suggests that linezolid has an advantage over glycopeptides such as vancomycin and teicoplanin in the treatment of proven MRSA pneumonia^3–5,48,49^. At least four potential reasons underlie this effect. Firstly, concentrations in ELF can consistently exceed the MIC breakpoint (4 mg/L) needed for adequate antimicrobial activity^31,34,48,50^ with concentrations reported to stay above MIC levels 100% of the time^31^. Secondly, vancomycin is associated with renal toxicity and neutropenia^51^. Thirdly, protein synthesis inhibition by linezolid produces a ‘non-bacteriolytic’ action and reduces virulence factor expression^52–54^. Finally, linezolid may affect host cell functions and cytokine networks^41–46^. Indeed, a recent experimental infection model of MRSA pneumonia and clinical studies in community-acquired MRSA pneumonia suggest protective immunomodulatory effects of linezolid^55,56^. These studies lend support to the hypothesis that linezolid has ‘additional’ antimicrobial efficacy through mechanisms involving immunomodulation.

We accept that this study is not without limitations. Firstly, we did not investigate the effects of glycopeptides (such as vancomycin) on neutrophils. We did investigate vancomycin as a potential control early in this study but found that dosing was inconsistent especially when combined with C5a in the dysfunctional model and thus did not pursue this further. However, the effects of vancomycin on neutrophils have been well studied^57–60^, and it is interesting to note that dosing remains a difficulty following 60 years of use^61^. Secondly, this study used one strain of MRSA (VAP 39) from our previous studies^12^, but selected appropriately with justification from a choice of 6 strains (Table 1 and supplementary Fig. 1). This allowed the current study to focus on intricate cell biology. Future work will investigate these effects across the wider *S. aureus* species. Thirdly, we also undertook preliminary experiments with cellular inhibitors (e.g. cytochalasin D and MAP kinase inhibitors) as we have done previously^12,13,21,62^, however the use of three reagents (C5a, linezolid and inhibitor) resulted in overwhelming cell toxicity (95–100% cell death) and were discontinued. Fourth, studies using C5a to investigate its effects in assays of neutrophil function/dysfunction should take care in design as chemotactic agents can have contrasting effects and lead to neutrophil priming/de-priming responses^63–65^. Finally, studies such as this could be applied to the next generation of novel oxazolidinone and anti-staphylococcal agents, such as tedizolid, which has potential for treating MRSA infections^66^.

**Table 1:** VAP and non VAP MRSA strains.

Organism(s)	Strain	Islolated from	CFU/ml	VAP (+ / −)	Antibiotic MIC (μg/ml)	
					Linezolid	Vancomycin
*H. influenzae* *S. aureus* (MRSA)	VAP 025	BAL	10^4^ 10^3^	+	4 (S)	1 (S)
*S. aureus* (MRSA)	VAP 026	BAL	10^2^	−	4 (S)	0.5 (S)
*S. aureus* (MRSA)	VAP 032	ETA	10^6^	−	4 (S)	1 (S)
*H. influenzae* *S. aureus* (MRSA)	VAP 034	BAL + ETA	10^4^ 10^4^	+	4 (S)	1 (S)
*S. aureus* (MRSA)	**VAP 039**	**BAL**	**10**^**4**^	** +**	**4 (S)**	**1 (S)**
Aspergillus spp. *S.aureus* (MRSA)	VAP 040	BAL	10^2^ 10^3^	−	4 (S)	1 (S)

Six MRSA strains were used and clinically defined as VAP or non VAP from a previous study^12^. Column 1 shows isolates detected in the original study with MRSA isolated and sub-cultured to produce the work in this manuscript. The strain used throughout this study, VAP39, is highlighted in bold.

To conclude, this study confirms that linezolid has immunomodulatory properties that protect human neutrophils from injury and provides insight into its mode of action beyond a basic antibiotic. These results will go some way in explaining why the therapeutic/treatment effect of linezolid is greater than its antimicrobial activity.

## Materials and methods

All methods were carried out in accordance with relevant guidelines and regulations.

### Bacterial strains and determination of MIC

Six clinical MRSA isolates obtained from bronchoalveolar alveolar fluid (BAL) in our previous studies were used (Table 1)^12,13^, and methicillin-sensitive *S. aureus* Cowan 1 was used as a control strain. Linezolid and vancomycin MICs were determined by using the limiting dilution method. Briefly, MRSA isolates were grown in tryptic soy broth ((TSB); Becton Dickinson, Cockeysville, USA)) overnight at 37 °C, then washed and resuspended to an OD_600_ = 0.1 (~ 1 × 10^7^ cfu/ml). MRSA isolates (5 × 10^5^ cfu) were then added to a 96-well plate containing a dilution series of linezolid (0.25–256 mg/L final) in TSB and incubated overnight at 37 °C. Plates were examined the following day and the lowest concentration of linezolid inhibiting visible growth determined. Antimicrobial sensitivity was compared to current EUCAST guidelines^19^.

### Selection of MRSA strain VAP 39

To select an appropriate MRSA strain for these studies all six MRSA isolates from our previous studies were tested for their sensitivity to linezolid and vancomycin (Table 1). Strains were isolated from the bronchoalveolar alveolar fluid (BAL) of patients, with 3 coming from patients with bacterial growth > 10^4^ colony-forming units/ml (CFU/ml) of lavage fluid, and 3 with growth below this conventional cut-off for the diagnosis of VAP. All strains had consistent MIC values for linezolid (4 μg/ml) and for vancomycin (1 μg/ml) except VAP26 (0.5 μg/ml). Thus, all MRSA strains were sensitive to linezolid and vancomycin by EUCAST guidelines^19^. Further consideration (Table 1) of the specificity of the source (BAL only) confirmed VAP39 as the test isolate for further studies. In addition, VAP39 was appropriate for downstream leukocyte assays, as comparisons between VAP39, VAP26 and laboratory reference, methicillin-sensitive *S. aureus* Cowan 1 strain showed no differences in killing and phagocytosis assays (Supplementary Fig. 1A–D). Therefore, VAP39 was selected for use in the remaining experiments.

### Bacterial culture for functional assays

One colony of MRSA VAP39 was inoculated into TSB and incubated overnight at 37 °C*.* One millilitre of overnight culture was centrifuged at 9677* g* and the supernatant removed. Pellets were resuspended in 1 ml of Iscove’s Modified Dulbecco’s Medium (IMDM, Thermofisher) and washed once before measuring the OD_600_ and adjusted to OD_600_ = 0.1 (~ 1 × 10^7^ cfu/ml).

### Isolation of human neutrophils

Whole blood from healthy volunteers was isolated using the vacuette blood collection system (5–9 ml) on the day of the experiment. Volunteers gave written informed consent. The project (Reference 13/WA/0190) was reviewed and the procedures and protocols approved by the local research ethics committee, Wales REC 6 (E-mail: Wales.REC6@nhs.uk). Healthy volunteer neutrophils were isolated and purified as previously described^67^. Freshly drawn blood was collected into citrated (light blue tops) tubes and mixed by gentle inversion prior to centrifugation at 350* g* for 20 min. The platelet rich plasma was aspirated (for serum generation) and the leukocytes remaining in the cellular layer separated from red blood cells through dextran (1.25% final concentration) sedimentation. Leukocytes were removed with a Pasteur pipette and washed with warm saline, before centrifugation at 350* g* for 6 min. The leukocyte cell pellet was resuspended in 3 ml of 55% isotonic percoll (GE Healthcare). Then, a tube containing overlaid solutions of percoll was prepared comprising 3 ml each of 81%, 70% and the leukocyte suspension in 55% percoll. Then, percoll gradients were centrifuged at 720* g* for 20 min. Granulocytes were harvested at the 70/81% interface using a Pasteur pipette, washed in PBS without calcium and magnesium prior to centrifugation at 230* g* for 6 min. Granulocytes were resuspended in buffer appropriate for functional assay. Neutrophil were only used at > 95% purity. Neutrophils counted by trypan blue staining after treatment with C5a/linezolid alone or in combination had viabilities ranging from 90 to 98%.

### Generation of neutrophil dysfunction

Purified neutrophils were resuspended to 1 × 10^7^ viable cells/ml in IMDM, then diluted to 1 × 10^6^/ml in IMDM containing 3% autologous serum and recombinant human C5a (R and D Systems, Abingdon) at 1–100 ng/ml or untreated control^12^. Neutrophils were incubated by rotation (10 rpm) at 37 °C for 16 h prior to functional assays. Neutrophil viable counts were re-assessed by trypan blue exclusion and adjusted once again to 1 × 10^6^ viable cells/ml.

### Linezolid incubation conditions

Linezolid was used at concentrations (0.4–40 mg/ml) consistent with tissue and cellular levels of the drug in humans^31–34^. Three incubation strategies were used to study the effect of linezolid on neutrophils.

1. To study protection against injury: Linezolid (0.4–40 mg/L) was added 1 h prior to C5a injury and washed out before addition of C5a;
2. To study inhibition of injury: Linezolid (0.4–40 mg/L) was added in combination with C5a for 16 h;
3. To study restoration of function: Linezolid (0.4–40 mg/L) was added for 1 h following C5a incubation.

After each treatment, neutrophils were centrifuged at 300* g* for 5 min, the supernatant removed and cells gently re-suspended in appropriate buffer for functional assays. Linezolid was removed prior to all assays involving MRSA so that it would have no direct antimicrobial effect on the bacteria.

### Neutrophil functional assays

1. Phagocytosis and killing assays

MRSA (OD_600_ = 1) was pre-opsonised with 100% autologous serum for 30 min at 37 °C and then corrected to an OD_600_ = 0.1 in 3% autologous serum/IMDM. Treated (C5a and linezolid as above) and untreated neutrophils at twice normal concentration were exposed for 2 h to pre-opsonised MRSA (multiplicity of infection = 1). Incubation was carried out at constant rotation (10 rpm) at 37 °C. Then 80 μl of MRSA-infected neutrophils was used for cytospin preparations (300 rpm for 3 min), air dried and stained with Hemacolor according to the manufacturer’s instructions (Millipore Ltd, Watford). For a quantitative measure of phagocytosis, light microscopy was used to determine the number of neutrophils containing bacteria within phagosomes with the results expressed as a percentage (Supplementary Image 1 and 2). The remaining infected neutrophils were used to assess viable counts by gentle lysis in 0.1% Triton X100 for 1 min to release intracellular bacteria. Lysates were diluted, plated on TSB agar and incubated overnight at 37 °C prior to determination of viable counts.

2. Superoxide assay

Neutrophil superoxide was assayed by cytochrome c reduction assay as described previously^68^. Briefly, treated (C5a and linezolid) and untreated neutrophils were re-suspended in 1 ml Hank’s Balanced Salt Solution (HBSS; Thermofisher) with calcium at 1 × 10^7^/ml. Then in separate tubes 50 μl of neutrophils (i.e. 500,000 cells), 800 μl of cytochrome C (1.25 mg/ml, Sigma Aldrich) and 100 µl of phorbol myristol acetate (PMA, final concentration 100 nM; Sigma Aldrich) were combined and incubated at 37 °C for 15 min. The reaction was terminated by centrifuging at 300×*g* for 5 min at 4 °C, and the OD_550_ of the supernatant determined. The ‘nM of O_2_^−^ released per million cells’ was calculated from:

$$\frac{{{\text{Observed}}\,{\text{Peak}}\,{\text{Absorbance}}\; \times \;{47}.{6}\;\left( {{\text{extinction}}\;{\text{coefficient}}} \right)}}{{{\text{Number}}\;{\text{of}}\;{\text{million}}\,{\text{cells}} \times 0.{5}}}$$

3. Transmigration assay

The ability of neutrophils to migrate was assessed by using a Transwell transmigration assay system (pore size 3 μm supplied by VWR, Lutterworth, UK), where IL-8 (1–100 ng/ml) was placed in the lower chamber and 1 × 10^5^ treated (C5a and linezolid as above) or untreated neutrophils added to the top chamber. Following 90 min incubation at 37 °C the membrane of the top chamber was wiped very gently with a cotton bud to remove cells that had not migrated and fixed for 10 min in 100% methanol. The Transwell filter was then stained with Hemacolor, cut from the Transwell casing and mounted on a microscope slide with a drop of DPX (Sigma Aldrich, Gillingham). When dry, transmigration was quantified by counting the number of neutrophils (Supplementary Image 3) using a light microscope (100 × oil immersion).

## Statistical analysis

Data are presented as mean ± standard error of the mean (SEM) as assessed in Excel. Plots were generated using GraphPad Prism software (V5.00 for Windows, GraphPad Software, San Diego California USA). Data were subjected to a Shapiro–Wilk normality test and then a one way-analysis of variance (ANOVA) with Tukey’s post-hoc test. In Supplementary Fig. 1 a two-way ANOVA and Bonferonni post-hoc test was used. Differences between treatment groups were considered statistically significant if *p* < 0.05.

## Electronic supplementary material

Below is the link to the electronic supplementary material.

Supplementary Information.
