# Supplementary material for: Contrasting effects of linezolid on healthy and dysfunctional human neutrophils: reducing C5a-induced injury
Source: Sci Rep. 2020 Oct 2;10:16377. doi: 10.1038/s41598-020-72454-0 (PMC7532177; doi:10.1038/s41598-020-72454-0)

SUPPLEMENTARY DATA (Figures and Images)

Contrasting effects of linezolid on healthy and dysfunctional human neutrophils: reducing C5a-induced injury.

[1]Stephen J. Evans, [1]Aled E. L. Roberts, [2]Andrew Conway Morris, [3]A. John Simpson, [1]Llinos G. Harris, [1,4]Dietrich Mack, [1]Rowena E. Jenkins and [1]Thomas S. Wilkinson

[1]Microbiology and Infectious Disease, Institute of Life Science, Swansea University Medical School, Singleton Park, Swansea, SA2 8PP
[2]Division of Anaesthesia, Department of Medicine, School of Clinical Medicine, University of Cambridge, Box 93, level 4, Addenbrooke's Hospital, Cambridge Biomedical Campus, Hills Road, Cambridge CB2, 0QQ
[3]Institute of Cellular Medicine, Medical School, Newcastle University, Newcastle upon Tyne NE2 4HH
[4]Bioscientia Labor Ingelheim, Institut für Medizinische Diagnostik GmbH, Konrad-Adenauer-Str. 17, 55218 Ingelheim, Germany

Corresponding author:

Thomas S. Wilkinson

Microbiology and Infectious Disease
Institute of Life Science
Floor 1, Room 137,
Swansea University Medical School,
Singleton Park,
Swansea, SA2 8PP

Tel: +44 1792 295018
E-mail: t.s.wilkinson@swansea.ac.uk

Key Words: Neutrophil dysfunction, linezolid, methicillin-resistant *Staphylococcus aureus*, ventilator associated pneumonia

Running Title: The effects of linezolid on human neutrophils

Supplementary Figure 1: Neutrophil killing and phagocytosis of VAP, non VAP and *S. aureus* Cowan 1 (SAC)

Purified neutrophils were exposed to A) *S. aureus* Cowan 1 (red); B) VAP 26 (green); and C) VAP 39 (blue). Killing is expressed as viable counts of MRSA (cfu/ml). D) Phagocytosis expressed as the percentage of neutrophils containing MRSA. Data are expressed as the mean ± SEM of 4 separate donors. *represents a significant difference between viable counts of MRSA at t=0 vs t=2 with neutrophils present and is true for all three isolates. ** represents a significant difference in phagocytosis between neutrophils at t=0 vs t=1, 2 or 4 for each strain measured.

Supplementary Figure 2: Effects of linezolid on functional and dysfunctional neutrophil killing

Linezolid (0, 0.4, 4 and 40 mg/L) was either A) pre-incubated or B) post-incubated for 1 hour compared to the C5a incubation period. Killing expressed as viable counts of MRSA (cfu/ml). No significant differences were identified White bar represents viable MRSA at t=0. Hatched bar represents viable MRSA at t=2 without neutrophils. Grey and black bars represent neutrophils at t=2 without and with C5a respectively.

Supplementary Figure 3: Effects of linezolid on functional and dysfunctional neutrophil phagocytosis

Linezolid (0, 0.4, 4 and 40 mg/L) was either A) pre-incubated or B) post-incubated for 1 hour compared to the C5a incubation period. Phagocytosis expressed as the percentage of neutrophils containing MRSA. No significant differences were identified. Grey and black bars represent neutrophils at t=2 without and with C5a respectively.

Supplementary Figure 4: Viability of neutrophils following exposure to vancomycin and linezolid and assessed using alamar blue

Purified neutrophils were exposed to vancomycin (2-128 mg/L), linezolid (4-40 mg/L) or triton (0.1-10% w/v) as positive control for A) 1 hours or B) 16 hours to mimic pre/post exposure or co-exposure protocols respectively. Results are metabolic activity (reducing power) expressed as a percentage of the untreated time point control. Data is expressed as the mean ± SEM of 3 separate donors. *represents a significant difference between untreated neutrophils and 0.1, 1 or 10% triton treated neutrophils.

Supplementary Figure 5: Effects of linezolid on functional and dysfunctional neutrophil IL-8 induced transmigration.

Functional and dysfunctional neutrophils were either A) pre-incubated or B) post-incubated with linezolid (0, 0.4, 4 and 40 mg/L) compared to the C5a incubation period, then transmigration assays were carried out and are expressed as the number of cells counted per high power field. Grey and black bars represent functional and dysfunctional neutrophils respectively. *represents a significant difference between the transmigration of functional neutrophils with and without IL-8. **represents a significant difference between the transmigration of functional neutrophils treated with and without IL-8.

Supplementary Figure 6: Effects of linezolid on functional and dysfunctional neutrophil respiratory burst

Functional and dysfunctional neutrophils were either A) pre-incubated or B) post-incubated with linezolid (0, 0.4, 4 and 40 mg/L) for 1 hour compared to the C5a incubation period, followed by a respiratory burst assay. Grey and black bars represent functional and dysfunctional neutrophils respectively. *represents a significant difference between PMA treated and untreated functional neutrophils (grey bars). # represents a significant difference between PMA treated and untreated dysfunctional neutrophils (black bars)

Supplementary Image 1: Phagocytosis assay
To quantify levels of phagocytosis the number of neutrophils with and without phagocytosed MRSA were counted and the results expressed as % phagocytosis.

Supplementary Image 2: Effect of C5a on phagocytosis
Representative images of neutrophils treated with and without C5a (100ng/ml).

Supplementary Image 3: Transmigration assay
To quantify transmigration Transwell membranes were removed and stained with Heamacolor prior to counting neutrophils per field of view. Representative images show transmigration with and without IL-8 (100ng/ml).

Supplementary Methods

Alamar blue assay

The alamar blue assay is based on resazurin-based reagents that function as cell health indicators by using the reducing power of living cells to quantitatively assess ‘metabolic activity’ as a measure of viability. Purified neutrophils (3 x 10^5) were added in a volume of 100µl to each well of a 96 well plate, followed by antibiotics in a volume of 100µl (at 2X final concentrations). Final concentrations of antibiotics were vancomycin (2-128 mg/L), linezolid (4-40 mg/L) and positive control triton (0.1-10% w/v). Then 25µl of alamar blue reagent was added to the wells and incubated at 37°C in a 5% CO_2 environment for up to 18 hours. At 1 and 16 hours the plate was read on a BMG Omega plate reader at 570nm (BMG, Offenburg, Germany).

Supplementary Figure 1

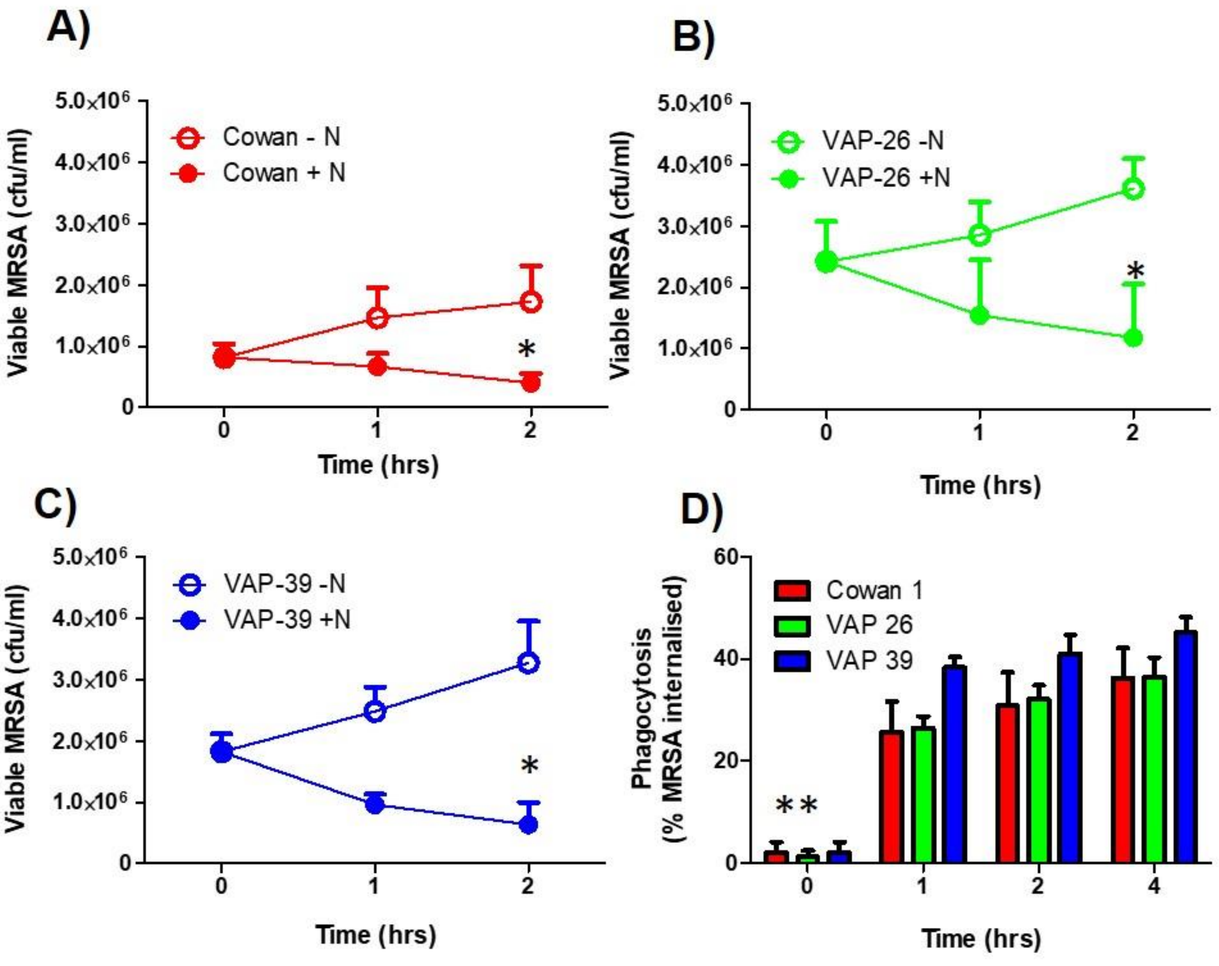

Supplementary Figure 2

A)

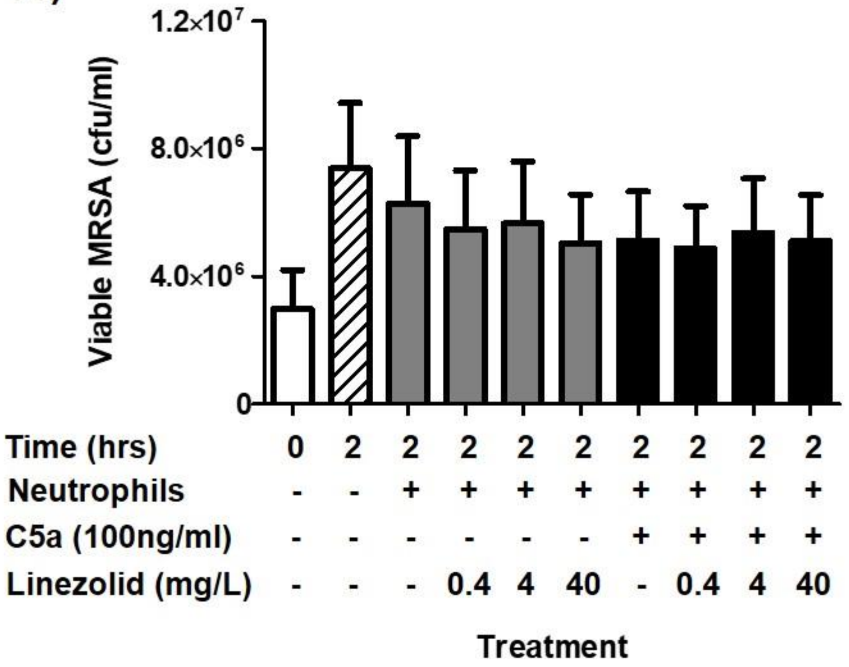

B)

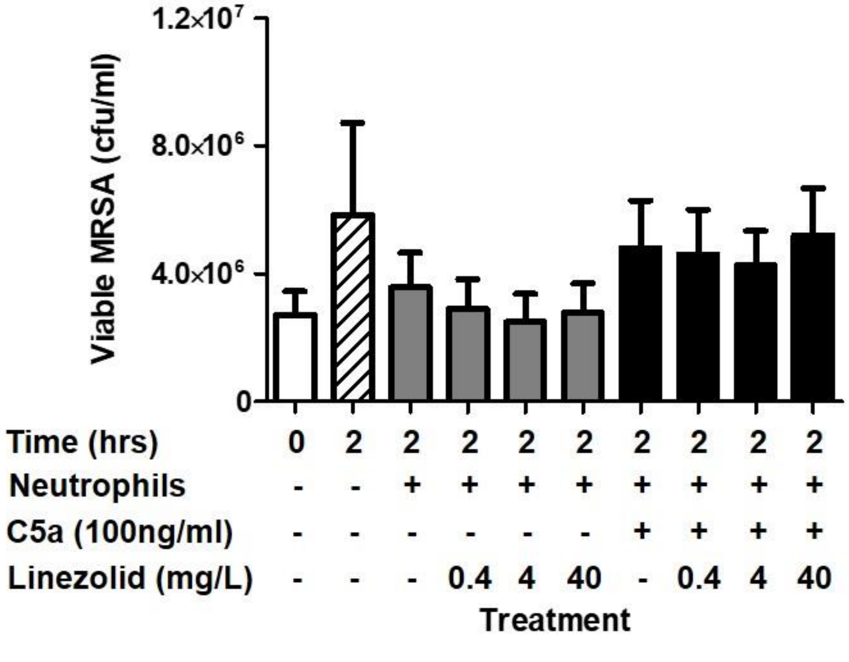

Supplementary Figure 3

A)

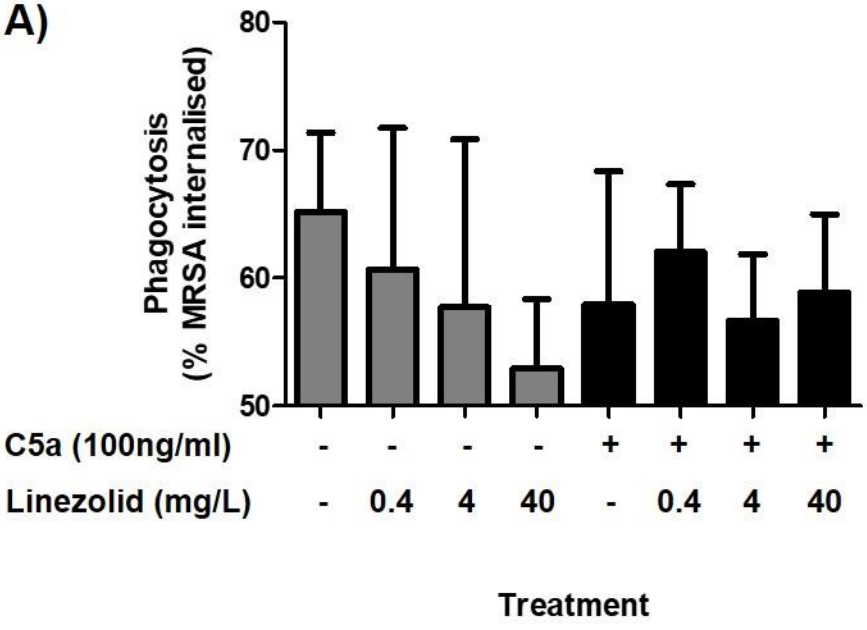

B)

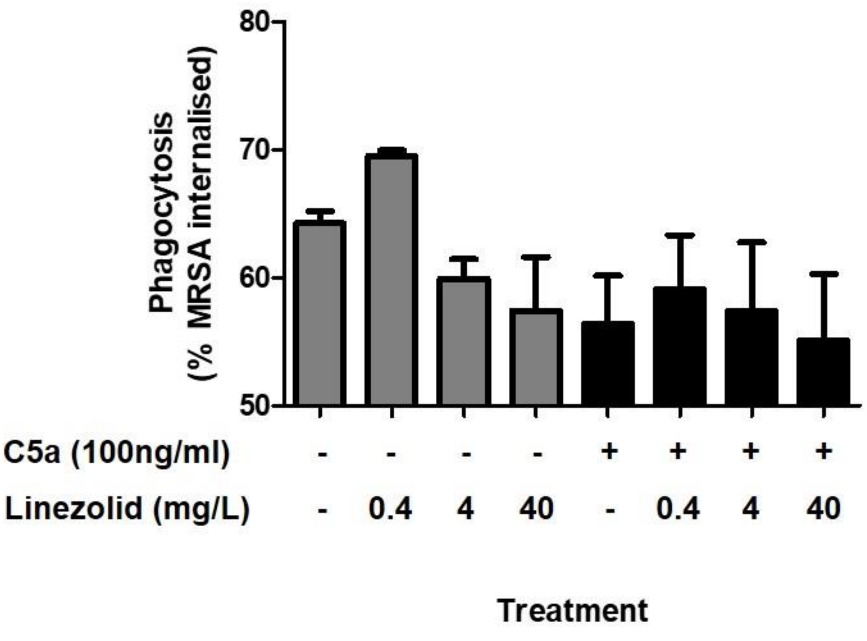

Supplementary Figure 4

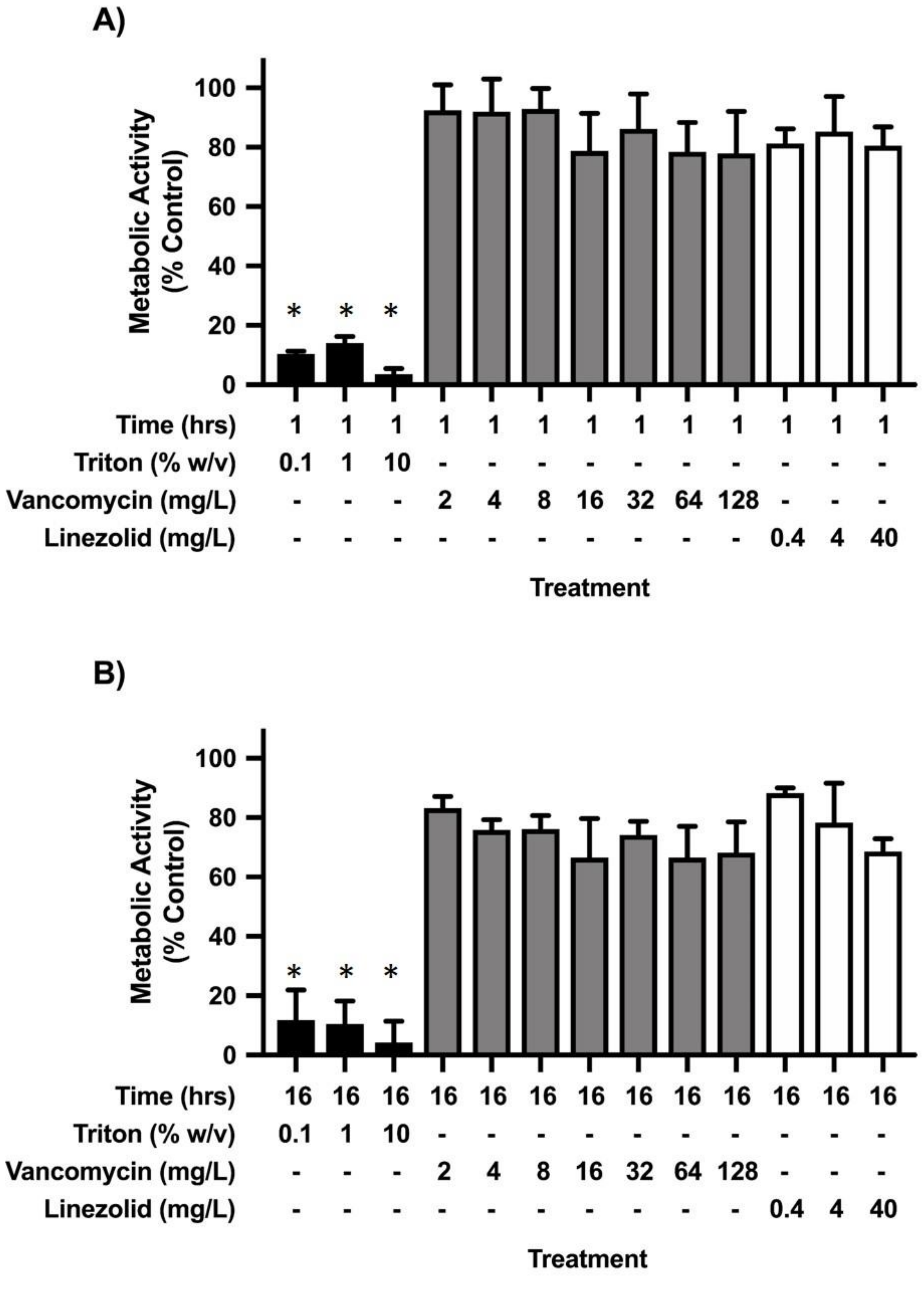

Supplementary Figure 5

A)

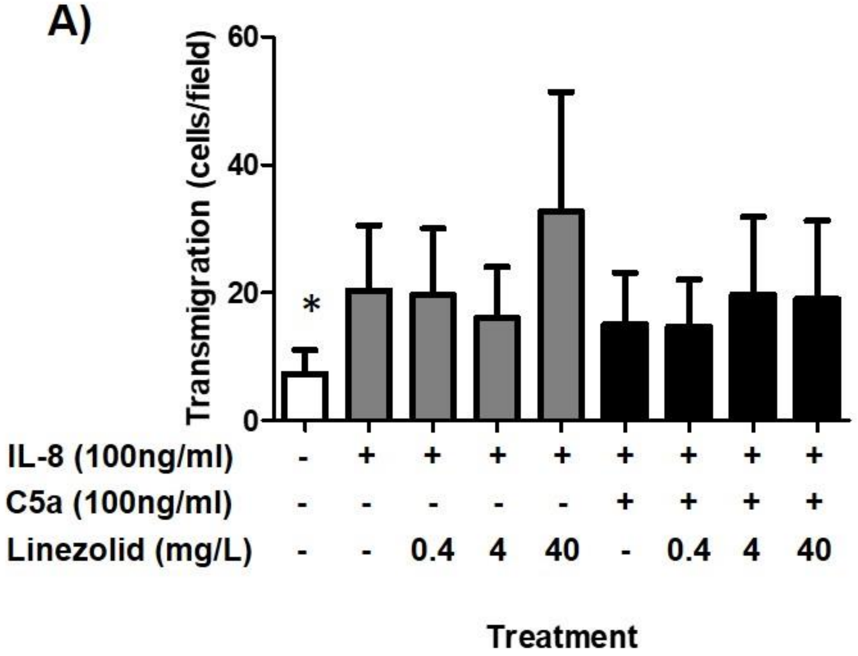

B)

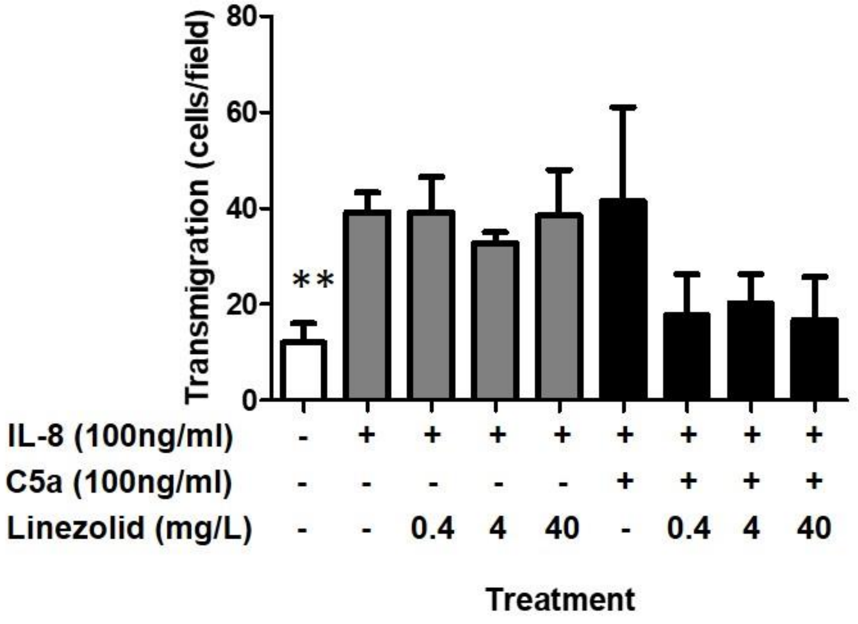

Supplementary Figure 6

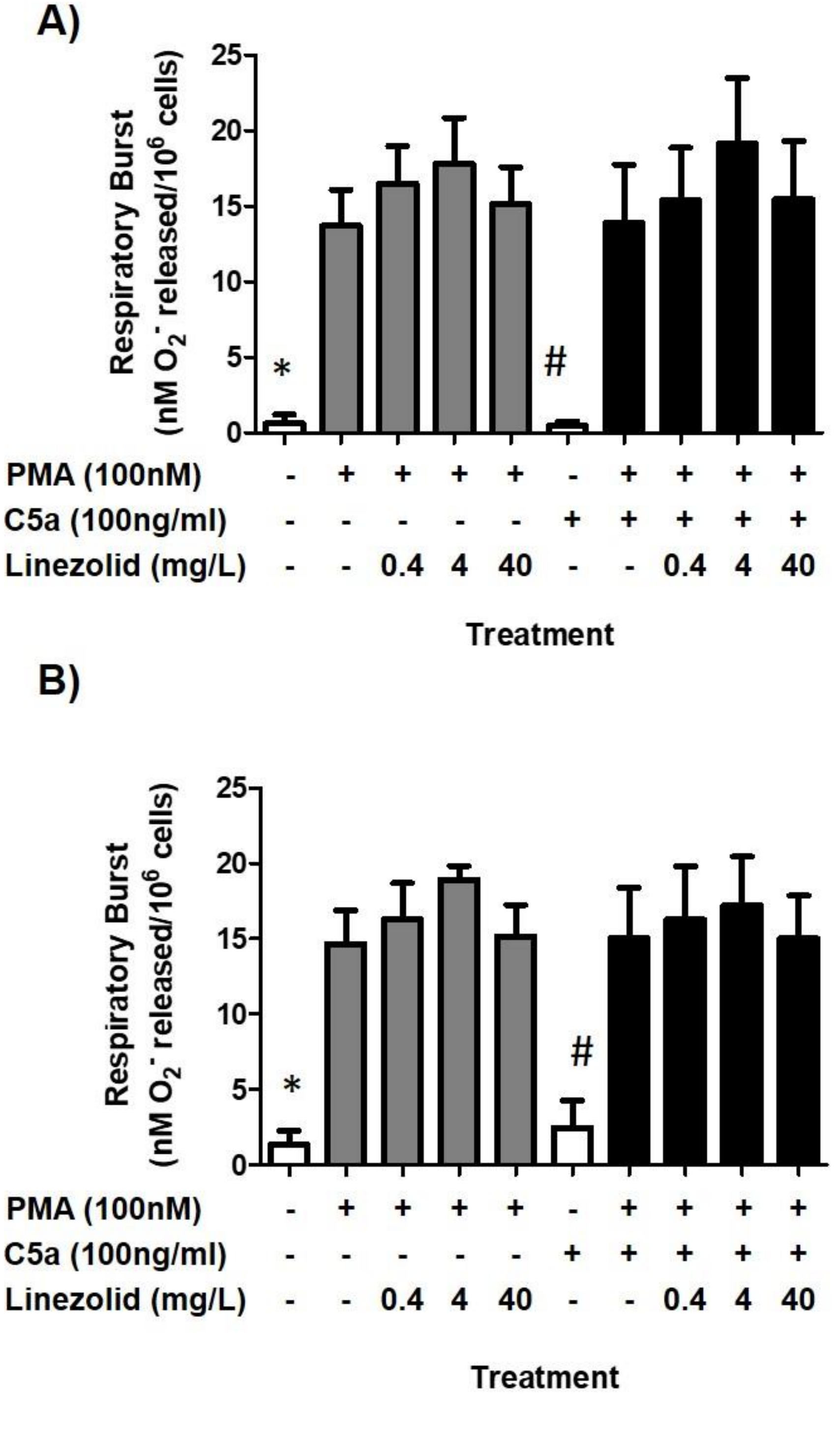

Supplementary Image 1

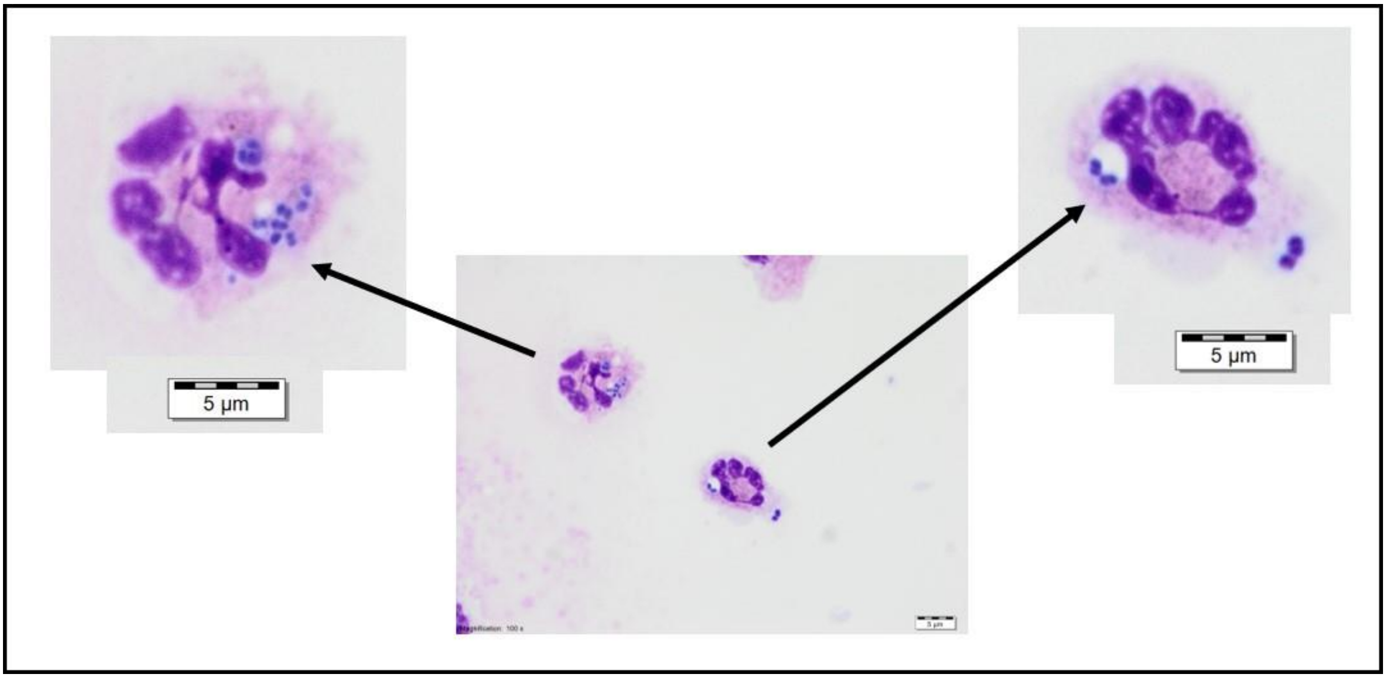

Supplementary Image 2

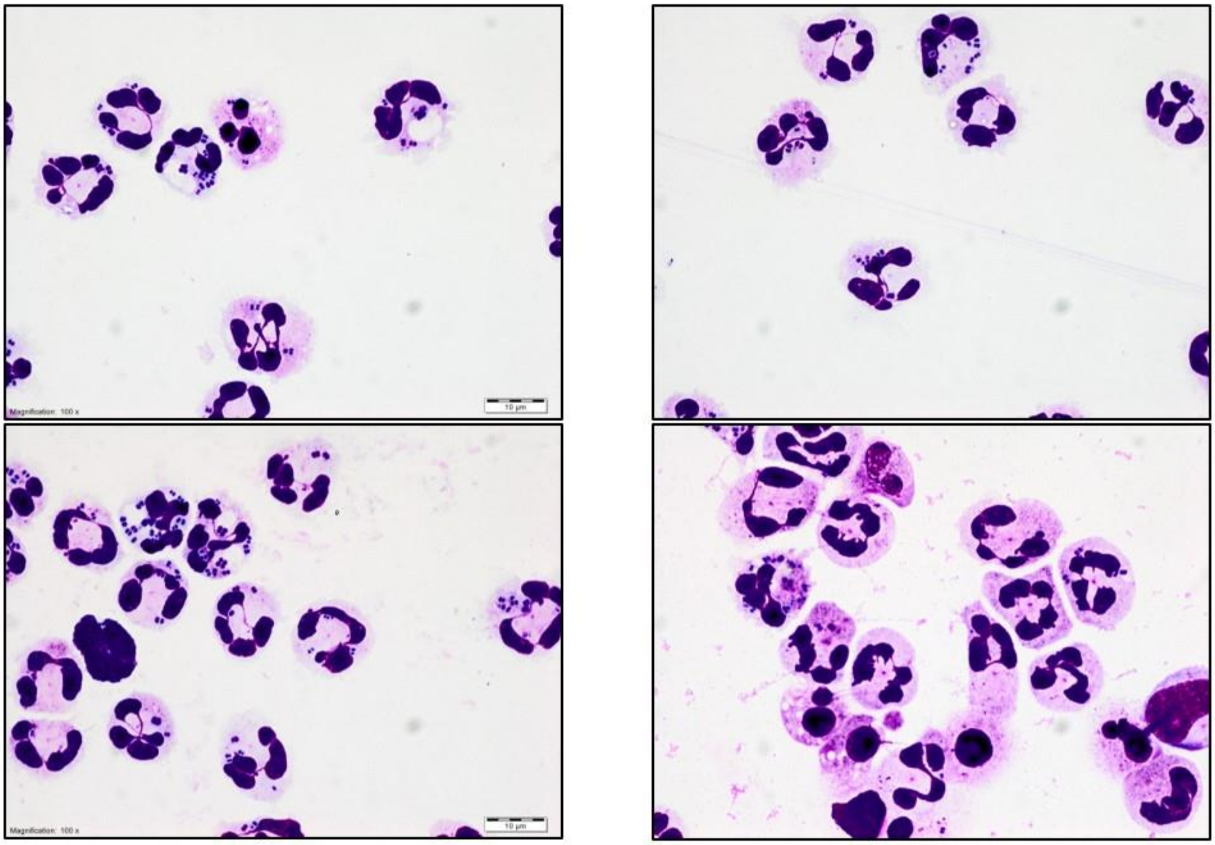

-C5a

+C5a (100ng/ml)

Supplementary Image 3

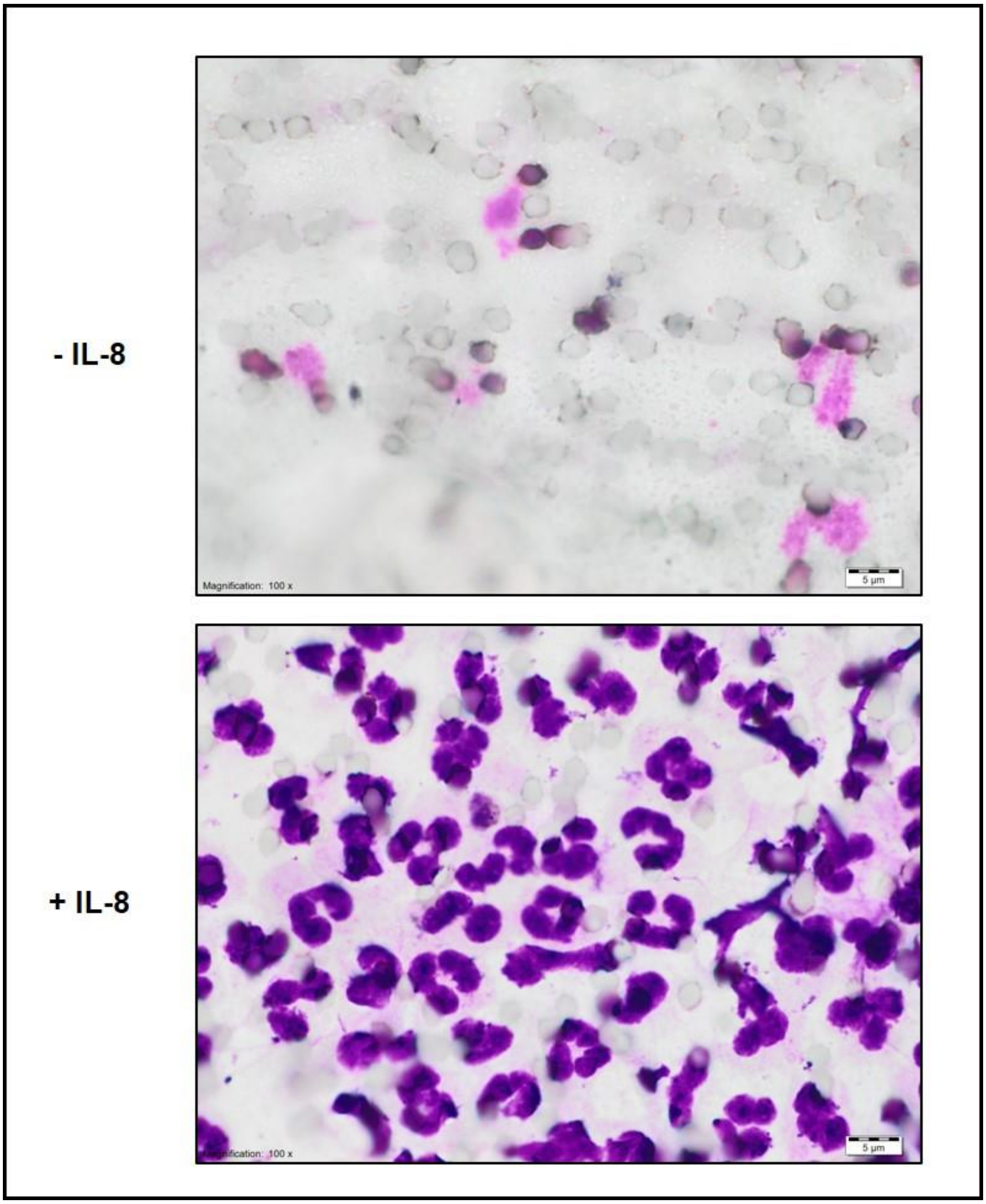

Supplement: Supplementary file 1 — Supplementary Information. [file 41598_2020_72454_MOESM1_ESM.pdf]
